# Resistance Breakdown of a Membraneless Hydrogen–Bromine Redox Flow Battery

**DOI:** 10.1021/acssuschemeng.2c02169

**Published:** 2022-09-21

**Authors:** Daniel Alfisi, Amit N. Shocron, Robert Gloukhovski, David A. Vermaas, Matthew E. Suss

**Affiliations:** † Faculty of Mechanical Engineering, Technion—Israel Institute of Technology, Haifa 3200003, Israel; ‡ Department of Chemical Engineering, Delft University of Technology, Delft 2628, The Netherlands; § Wolfson Department of Chemical Engineering, Technion—Israel Institute of Technology, Haifa 3200003, Israel; ∥ Grand Technion Energy Program, Technion—Israel Institute of Technology, Haifa 3200003, Israel

**Keywords:** Redox Flow Batteries, Energy Storage, Electrical Grid, Hydrogen−Bromine Battery, Electrochemical Impedance Spectroscopy

## Abstract

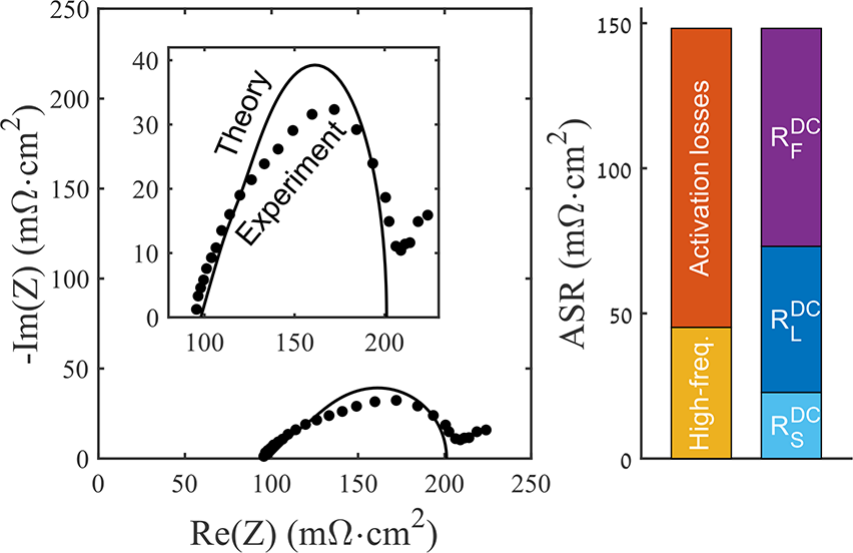

A key bottleneck to society’s transition to renewable energy is the lack of cost-effective energy storage systems. Hydrogen–bromine redox flow batteries are seen as a promising solution, due to the use of low-cost reactants and highly conductive electrolytes, but market penetration is prevented due to high capital costs, for example due to costly membranes to prevent bromine crossover. Membraneless hydrogen–bromine cells relying on colaminar flows have thus been investigated, showing high power density nearing 1 W/cm^2^. However, no detailed breakdown of resistance losses has been performed to-date, a knowledge gap which impedes further progress. Here, we characterize such a battery, showing the main sources of loss are the porous cathode, due to both Faradaic and Ohmic losses, followed by Ohmic losses in the electrolyte channel, with all other sources relatively minor contributors. We further develop and fit analytical expressions for the impedance of porous electrodes in high power density electrochemical cells to impedance measurements from our battery, which enabled the detailed cell resistance breakdown and determination of important electrode parameters such as volumetric exchange current density and specific capacitance. The insights developed here will enable improved engineering designs to unlock exceptionally high-power density membraneless flow batteries.

## Abstract 2

Membraneless hydrogen−bromine batteries are promising toward high power density storage. We here identify major resistance losses to accelerate their development.

## Introduction

Over the past several decades, energy consumption has been growing significantly in every sector and source,^1^ driving interest in renewable energy with minimal greenhouse gas emissions. However, penetration of renewable energy sources is limited, with a major reason being that such sources are often intermittent, such as solar and wind energy.^2^ Thus, grid-scale energy storage is required to time-shift the generated energy and obtain a more uniform power output from a renewable energy plant. One promising technology for grid-scale energy storage are redox flow batteries (RFBs), which are distinct from other batteries such as lithium ion as the reactants are stored in external tanks and circulated through the battery cell.^3−5^ RFBs modular design allows for a spatial decoupling of energy stored (in tanks) and power delivery (in the battery), which allows for potentially inexpensive upscaling to grid-scale energy storage of MWh capacity. RFBs tend to have lower energy density than the lithium ion battery, but can achieve higher power density, more charge/discharge cycles, and utilize less-expensive and earth-abundant reactants.^6−9^

Different RFB chemistries have been investigated toward the goal of commercialization with all-vanadium and zinc–bromine flow batteries generally the most commercially developed RFBs. Zinc–bromine flow batteries have been commercial for over a decade, with installations which can deliver up to 2 MW.^10^ Large-scale vanadium redox flow battery have been installed, such as in China with a 5 MW/10 MWh plant, and 15 MW/60 MWh in Japan.^11,12^ Other promising chemistries involving halides are at the lab-scale or in the early stages of commercialization, which include hydrogen–bromine,^8,13,14^ quinone-bromine,^15,16^ membraneless multiphase flow zinc–bromine,^17,18^ and zinc-iodide chemistries.^19,20^ Hydrogen–bromine is considered highly promising due to relatively low-cost reactants, fast electrochemical reaction kinetics, no metal catalysts for the bromine electrode, and exceptionally conductive electrolytes (>700 mS/cm).^21^ During discharge, H_2_ and Br_2_ form HBr as the reaction product, and this reaction is reversed during charging:
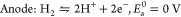
1
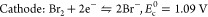
2
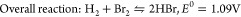
3

One of the major challenges in RFBs, preventing widespread adoption, is relatively high system capital costs. One strategy investigated for reducing battery cost per power is removing the membranes, as these can be responsible for up to 40% of the cell cost^22−24^ and can lead to issues such as membrane dehydration, increased cell Ohmic resistance, and a shortened lifetime.^25^ To maintain reactant separation without a membrane, several cell architectures and operational strategies have been investigated, such as use of colaminar flows or multiphase flows.^17,18,26,27^ Braff et al. proposed and studied a laminar hydrogen–bromine membraneless cell, relying on colaminar flows of hydrobromic acid and a hydrobromic acid/bromine mixture.^14,26^ This battery operated at a low Reynolds number yet large Péclet numbers to mitigate bromine transport into the hydrobromic acid stream and achieved a power density of nearly 0.795 W/cm^2^. Suss et al. implemented hierarchical flow-through porous cathodes in membraneless hydrogen–bromine batteries to enable higher current capability while minimizing crossover,^28^ achieving a room temperature power density of 0.925 W/cm^2^ and a current density of 3 A/cm^2^. Membrane-based hydrogen–bromine RFBs have achieved up to 1.46 W/cm^2^ at room temperature, and thus, membraneless cells could potentially surpass this power density as membrane conductivity is nearly an order of magnitude lower than that of 3 to 5 M HBr electrolyte.^7^ However, to date, the sources of voltage losses in such membraneless hydrogen–bromine cells have not been experimentally elucidated. This knowledge gap hinders further development and optimization of the membraneless hydrogen–bromine redox flow battery.

In this work, we fill the latter knowledge gap by providing a detailed resistance breakdown of a custom-built membraneless hydrogen–bromine RFB prototype. To enable such a breakdown, we developed an analytical expression for the impedance of a porous electrode of a high-power density cell, where the use of high conductivity electrolytes means the electrode’s solid-phase resistance cannot be neglected. We fit the theoretical impedance to experimental electrochemical impedance spectroscopy (EIS) results to extract key resistances and bromine electrode parameters. This is, to our knowledge, the first time such an impedance expression has been utilized to study electrodes in high power density flow battery cells. Overall, we find that the single biggest source of voltage loss is the porous bromine electrode, as it accounts for over 50% of the total cell area specific resistance (ASR). The second biggest is the electrolyte channel which accounts for ∼25% of cell ASR. Losses associated with other cell components were quantified but were relatively minor.

## Theory

**Figure 1:**
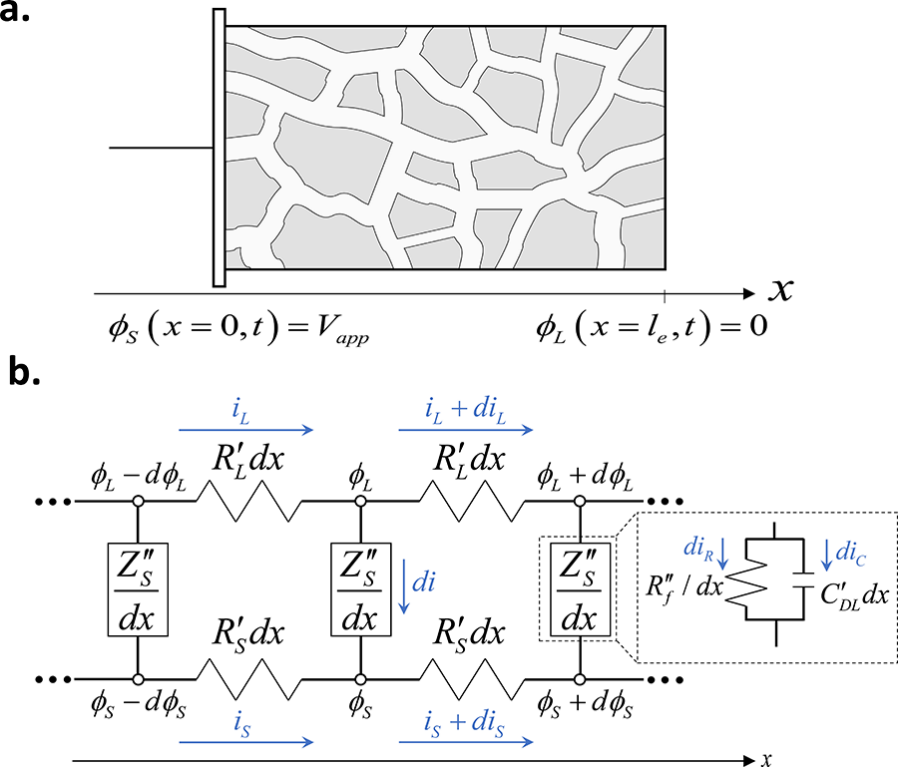
(a) Schematic of the porous electrode of a high power density redox flow battery. (b) Equivalent RC circuit used to describe the dynamics of the electrode shown in a. *i*_L_ and *i*_S_ are the current densities through the liquid and solid phases, respectively. Inset shows the interfacial impedance used in our model.

In order to develop insight and characterize the resistance of the porous bromine electrode of our membraneless design, we develop an expression for its impedance. Previous works investigating resistance losses in porous electrodes of high-power density flow batteries relied on 1D steady-state transport theory to interpret results,^16^ which allows for resistance breakdown from steady-state battery performance. However, other electrode properties which may help in interpreting electrode performance, such as electrode capacitance and so electrochemically active surface area, are more readily extracted from transient experiments, such as EIS. Further, use of EIS allows for convenient experimental linearization of the Faradaic resistance, which potentially allows for more accurate extraction of kinetic parameters. We here follow the general approach presented by De Levie,^29,30^ and describe the porous electrode using a transmission line model with resistive and capacitive elements, schematically shown in Figure 1. While many previous works used transmission line circuits to represent porous battery and flow battery electrodes, generally the solid phase electric resistance was justifiably neglected.^31,32^ However, in high power density flow batteries, such an assumption must be relaxed due to the high electrolyte ionic conductivity.^16,33^ Other assumptions invoked here are typical for transmission line models, including assuming spatially constant properties of the porous electrode, and negligible spatial variations of ion concentrations.^34^ The governing equations for this circuit model, resulting from the application of Ohm’s law across a differential element together with current conservation, are
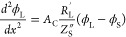
4
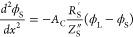
5Here, ϕ_L_ is the liquid potential, ϕ_S_ is the solid potential, *A*_C_ is the electrode cross-section area, *R*_L_^′^ is the liquid-phase resistance per unit length, *R*_S_^′^ is the solid-phase resistance per unit length, and *Z*_S_^″^ is the distributed impedance of the solid/liquid interface, relating the local potential difference and the local current density across the interface. The boundary conditions include setting the potential of the liquid at the pore inlet, where *x* equals the electrode thickness, *l*_e_ (see Figure 1a), to zero, the potential at the current collector to the applied potential *V*_app_, zero electric current through the liquid phase at the electrode/current collector interface, and zero electric current through the solid phase at the pore inlet:
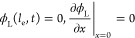
6
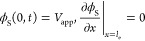
7

The problem given by eqs 4–7 was previously solved by Huang et al. and applied to polymeric electrolyte fuel cells where poor conductivity of the solid phase leads to non-negligible solid-phase resistance.^34^ We here instead focus on porous electrodes of high power density redox flow batteries where both solid and liquid phases are highly conductive. As given in Huang et al., the solution to eqs 4–7 results in the following impedance expression for a porous electrode with non-negligible solid-phase resistance:^34^
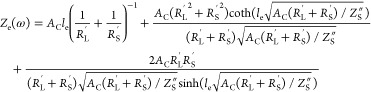
8Here, *Z*_e_ is the porous electrode impedance and ω is the angular frequency of the applied voltage or current. We consider the specific case of a distributed parallel RC circuit at the solid/liquid interface of the pore, capturing both electric double layer charging and Faradaic reactions at this interface, see the inset of Figure 1b. We assume that activation overpotential varies linearly with current, which is exact in the limit of small overpotentials ≪12.5 mV and reasonable for EIS experiments, so the interfacial impedance can be written as
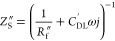
9Here, *R*_f_^″^ is the volumetric interfacial resistance to Faradaic reactions (units of mΩ·cm^3^), *C*_DL_^′^ is the double-layer volumetric capacitance (units of mF/cm^3^), and *j* is the imaginary unit. By substituting relation 9 into eq 8, we find the impedance of our porous electrode to be
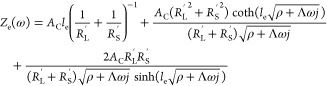
10where ρ ≡ *A*_C_(*R*_L_^′^ + *R*_S_^′^)/*R*_f_^″^ and Λ ≡ *A*_C_(*R*_L_^′^ + *R*_S_^′^)*C*_DL_^′^. For linearized activation overpotentials, we can express *R*_f_^″^ as^31^
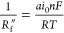
11where *n* is the number of electrons transferred per reactant olecule, *a* is the surface area per volume ratio, *i*_0_ is the exchange current density, *F* is Faraday's constant, *R* is the universal gas constant and *T* is the absolute temperature.

Next, we present the solution to eqs 4 and 5 for a potential drop between the solid and liquid phases, while invoking eq 9(34)
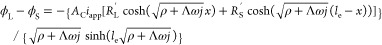
12where *i*_app_ is the applied current density. From eq 12, we can calculate *ϕ*_S_ by substituting it into eq 5 and applying boundary conditions 7. To probe local losses, we use the expression of *ϕ*_S_ to calculate electric current through the solid phase, *i*_S_, which results in
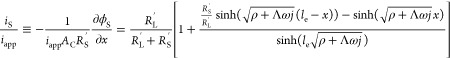
13

Using eq 13 at the DC limit where ω → 0, we can split the cathode Ohmic losses into three types, electronic, *R*_S_^DC^; ionic, *R*_L_^DC^; and Faradaic, *R*_F_^DC^. For constant current battery operation, where effectively ω → 0, the latter resistances can be used to describe Ohmic losses of the battery’s cathode due to solid phase, liquid phase, and Faradaic sources.^16^ For linearized activation overpotentials, we calculate these effective resistances to be
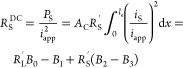
14
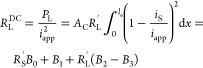
15
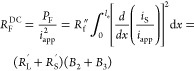
16Here, *P*_S_, *P*_L_, and *P*_F_ are the power dissipated by electronic, ionic, and Faradaic processes, respectively, and *B*_0_, *B*_1_, *B*_2_ and *B*_3_ are given by
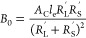
17
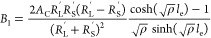
18
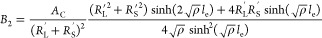
19
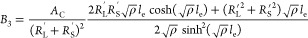
20

## Experimental Methods

**Figure 2:**
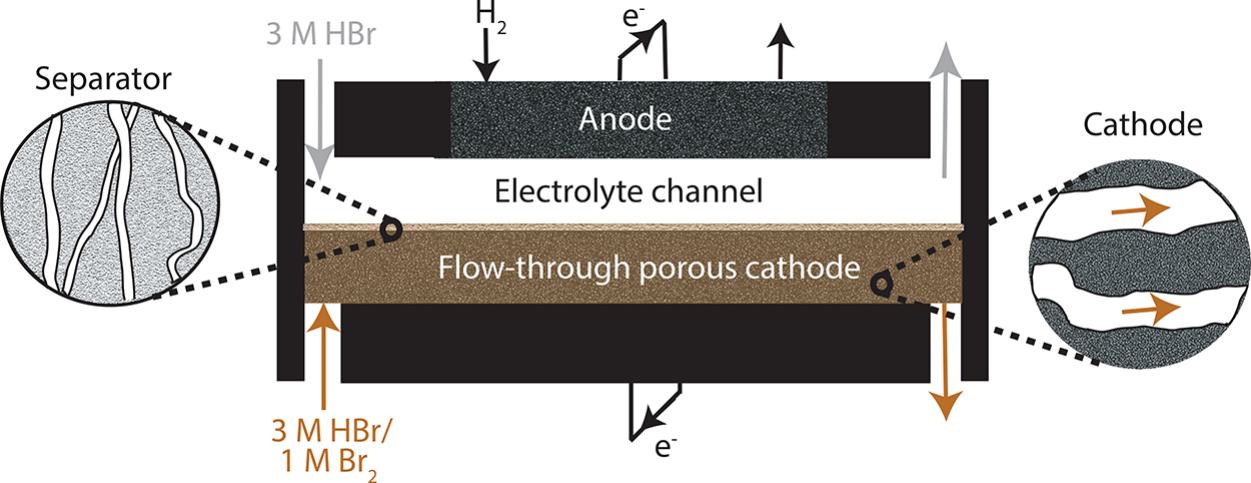
Schematic of the membraneless hydrogen–bromine redox flow battery used in this work.

**Figure 3:**
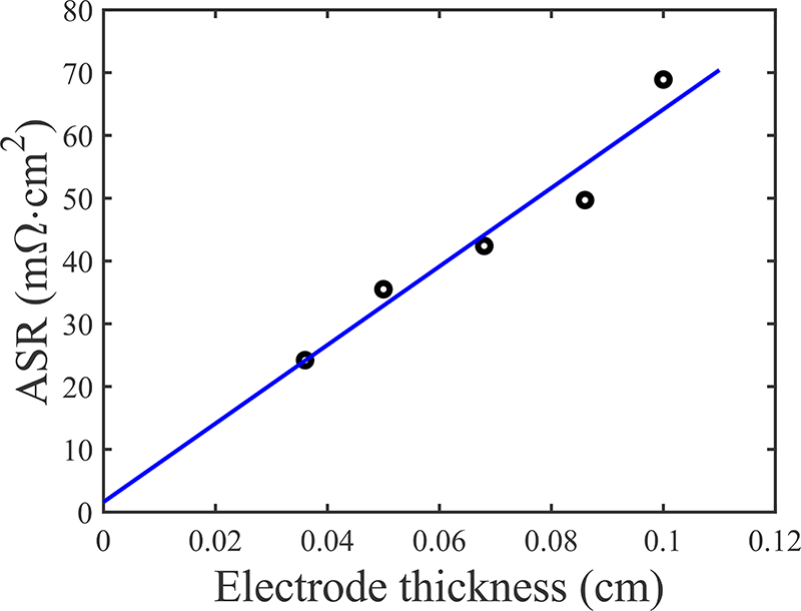
Measurements of the dry, through-plane area specific resistance (ASR) of our oxidized Sigracet 29AA cathode material, versus electrode thickness.

We developed a custom-built membraneless H_2_–Br_2_ battery, based on that described in Suss et al.,^28^ with two liquid flow channels (Figure 2). The electrolyte channel height was 0.65 mm, and the adjacent catholyte channel containing the porous cathode was 0.75 mm thick (Figure 2). The electrode used for the bromine half-reaction was placed in the catholyte channel, to form a flow-through porous cathode. The cathode consisted of six layers of Sigracet 29AA with an initial porosity of 80% and initial thickness of 180 μm for each layer. The carbon papers were pretreated via oxidation in the air at 500 °C for 1 h, which also reduced their thickness to about 155 μm. The cathode compression was about 24%, an optimum value as identified by Tucker et al.,^35^ but was uncompressed within the active area as the membraneless design had a liquid-only layer in the active area (electrolyte channel, Figure 2). Measurements of the dry through-plane resistance of the 24% compressed oxidized Sigracet 29AA papers were performed in a dedicated four-electrode impedance cell. The cell was comprised of two PVDF end plates between which two Ti sheet current collectors and two isomolded graphite plates for potential sensing sandwiched a 1 × 1.5 cm cathode material sample placed within a gasket of suitable thickness to maintain the desired compression. Potentiostatic EIS with a 10 mV peak-to-peak amplitude and 100 kHz to 100 mHz frequency range was applied to obtain the high frequency intercept of the impedance with the real axis, which was used as the resistance value. The best-fit curve to the measured dry resistance versus electrode thickness data of Figure 3 shows a slope of 625.4 mΩ·cm and a *y* intercept representing contact resistance between the SGL and graphite plate of 1.6 mΩ·cm^2^.

A microporous polypropylene separator, Celgard 3501 (Celgard Company, USA), was used as a dispersion blocker between the catholyte and electrolyte, to eliminate bromine crossover into the electrolyte channel via advection. The bromine entering the electrolyte channel from the catholyte by molecular diffusion is swept downstream before it reaches the anode, due to the high Péclet number of the electrolyte flow (Péclet of order 1000).^28^ The separator was nonselective toward ions, meaning it possesses negligible internal chemical charge and so does not act as an anion or cation exchange membrane, and thus the design is considered membraneless.^3,36−38^ The separator was hydrophilic, with a thickness of 25 μm and 55% porosity. For the anode, a commercial carbon cloth electrode with 0.5 mg/cm^2^ and 60% platinum was used (Fuel Cell Store, USA). Impervious and isomolded graphite plates of 3 mm thickness (Graphitestore, USA) were used as current collectors for the anode and cathode, respectively. We custom-milled an interdigitated flow field into the impervious graphite, for hydrogen distribution to the anode, with channels of 1 mm depth and width. Titanium sheets of 1 mm thickness were placed between the end plates and the graphite to ensure uniform current distribution in the battery. Polyvinylidene fluoride (PVDF) plates of 6 mm thickness were used as end plates. Hydrophobic gasketing material, including expanded PTFE gaskets and PTFE-coated glass fiber gaskets, were laser cut to form the liquid flow channels. The active area of the cell of 0.75 cm^2^ was defined by the open area in the electrolyte channel gasket. The battery cell was sealed via 14 M4 stainless steel bolts, plastic-coated to avoid short circuits, and sealed to a torque of 2.1 N·m. For some experiments, a quasi-reference electrode was inserted into the electrolyte channel, and the electrode used was a Pd wire of 125 μm diameter.

PTFE tubing (Bola, Germany) of 2 mm inner diameter was used to transport the electrolyte and catholyte solutions from external tanks to the battery. We used a 10 mL catholyte tank and 50 mL electrolyte tank, made from Teflon and polyethylene, respectively. Rigid PTFE tubing of 2 mm inner diameter was used in the peristaltic pump heads (Masterflex L/S digital, Cole-Parmer, USA). The electrolyte pumped through the electrolyte channel was 3 M HBr (Sigma-Aldrich, USA) with an ionic conductivity of 711 mS/cm, at a flow rate of 2 mL/min. This flow rate was chosen to enable an electrolyte channel pressure slightly above that of the catholyte channel, to prevent catholyte pumping into the electrolyte channel. A solution of 1 M Br_2_ and 3 M HBr was used as a catholyte and pumped at 1 mL/min through the cathode channel (Sigma-Aldrich, USA, 98% Br_2_ purity). The electrolyte and catholyte were pumped through the battery and then disposed, thus we utilized a single-pass operation mode. Hydrogen gas with a purity of 99.99% at a flow rate of 200 sccm flowed through the anode flowfield (MAXIMA, Israel). A potentiostat (Bio-Logic, France) in either two or three electrode configuration measured the voltage response for a set current density, with a dwell time of 60 s per current. For galvanostatic electrochemical impedance spectroscopy (GEIS) performed on the cell, we used a 10 mA current amplitude and a frequency range from 100 kHz to 100 mHz. The system was operated at room temperature and atmospheric pressure.

## Results and Discussion

**Figure 4:**
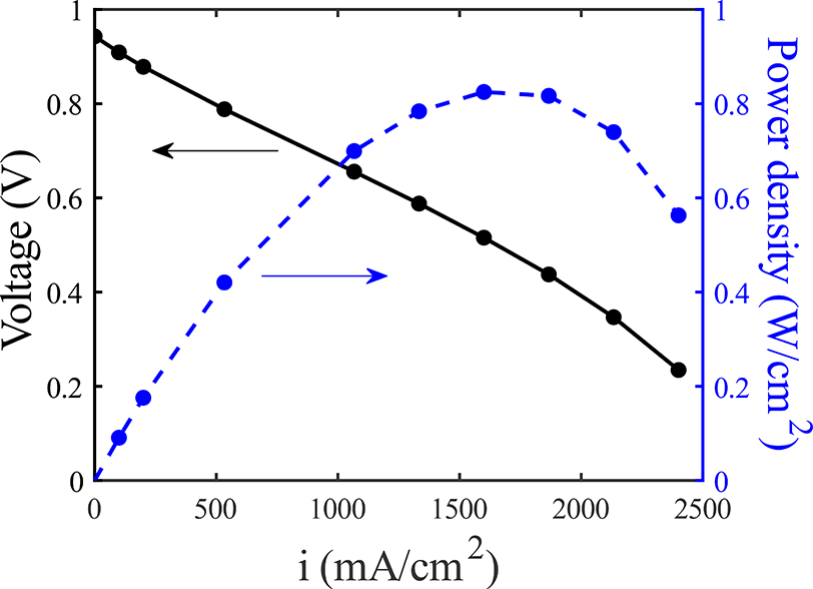
Measured discharge polarization curve and power density of our membraneless H_2_–Br_2_ prototype cell in two-electrode configuration.

In Figure 4, we show the results of a discharge polarization curve measurement on our prototype membraneless H_2_–Br_2_ flow battery. We observe an OCV of ∼0.94 V, followed by a linear region with voltage loss linearly proportional to current density to over 1 A/cm^2^ and evidence of mass transport losses at higher current densities. The slope of the linear region yields an ASR of ∼262 mΩ·cm^2^, and the maximum power density is achieved at 1.6 A/cm^2^ and is approximately 0.83 W/cm^2^. The OCV measured here and the linear behavior of the polarization curve are expected, and were seen also by Suss et al. for a similar membraneless H_2_–Br_2_ cell,^27^ although in the latter cell, discernible mass transport losses were not observed for currents at around 1 A/cm^2^. A linear discharge polarization curve was also attained by Chen et al., for a quinone-bromine RFB, but as described by the latter authors, in the linear region activation losses at the porous electrodes, both bromine and quinone, were significant.^16^

**Figure 5:**
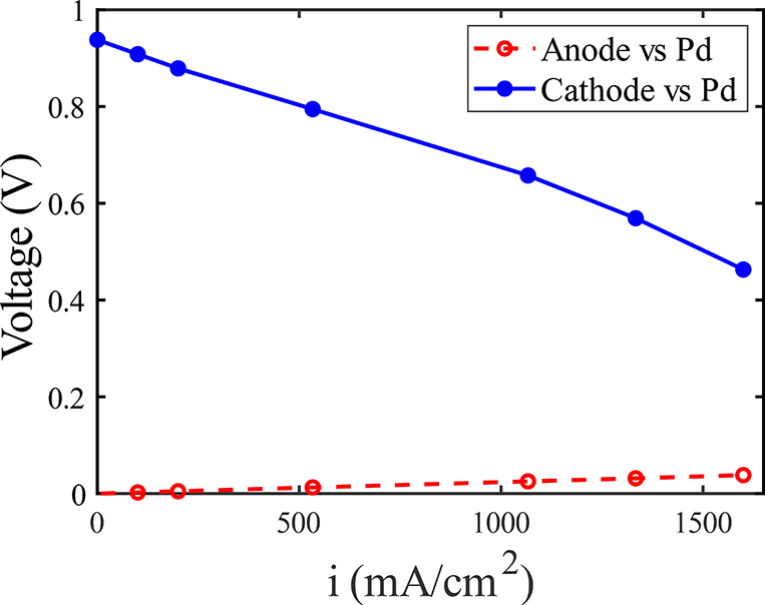
Measured discharge polarization curve in three-electrode configuration, showing the steady-state anode voltage (red circles) and cathode voltage (blue circles) versus a quasi-reference electrode during discharge.

We also performed discharge polarization curves on a separate build of the cell where a quasi-reference Pd electrode was placed into the electrolyte channel. This allowed measurement of the potential of the anode and cathode relative to the Pd electrode (Figure 5). The measured equilibrium voltage of the anode is near 0 V vs Pd and the cathode at ∼0.94 V vs Pd. As can be seen, the observed cell voltage losses are largely at the cathode side of the cell, with losses at the anode relatively small. For example, at the highest current tested in three-electrode configuration of 1.6 A/cm^2^, the cathode-side loss was ∼0.48 V relative to the equilibrium cathode voltage, while the anode shows <0.05 V loss relative to the equilibrium anode voltage. Further, these measurements confirm that the mass transport losses seen at currents >1 A/cm^2^ can be attributed to the cathode, and thus due to bromine starvation. We here use 1 M Br_2_ concentration and a catholyte flow rate of 1 mL/min, which can support a current density of up to 4.3 A/cm^2^ if all the bromine entering the porous cathode was electrochemically reduced in a single pass. Thus, it is expected to observe mass transport losses due to bromine starvation as we reach currents of >1 A/cm^2^.

**Figure 6:**
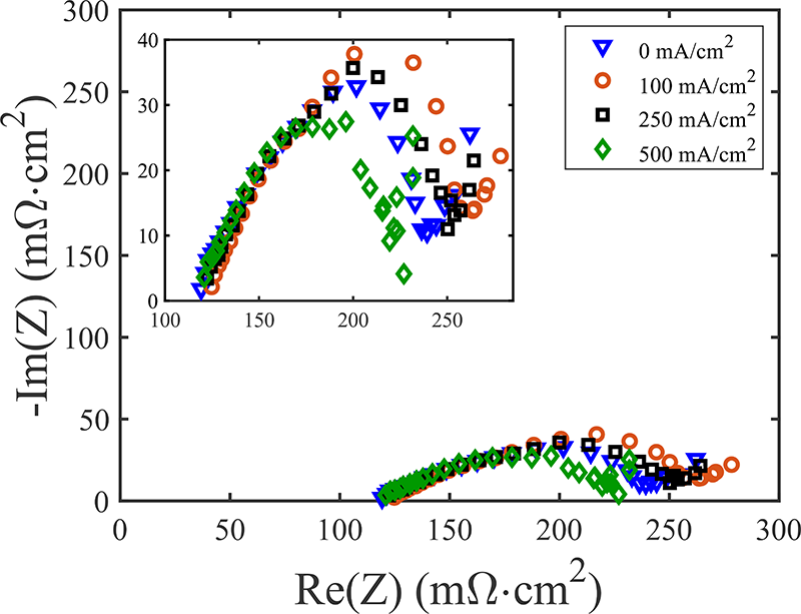
Measured impedance of our membraneless H_2_–Br_2_ battery, in the form of a Nyquist plot. Impedance is shown for various DC current values including 0 (blue triangles), 100 (red circles), 250 (black squares) and 500 mA/cm^2^ (green diamonds). Inset shows a zoom-in view of the measured impedance.

To further break down the resistances, we show results of GEIS measurements of our H_2_–Br_2_ membraneless battery cell in Figure 6, in the form of a Nyquist plot. Results are shown at various DC current densities, from 0 to 500 mA/cm^2^. For each current density, we see a high frequency intercept of ∼120 mΩ·cm^2^, followed by a distinct compressed semicircular feature, and apparent mass transport impedance at the lowest frequencies, between around 220–275 mΩ·cm^2^ on the real axis. We attribute the high frequency resistance to the summation of the resistances of elements in the cell which have no significant capacitive elements, which include the electrolyte channel and the Celgard separator, together with the high frequency response of the cathode, which is a parallel combination of the cathode solid and liquid-phase resistance.^16^ Based on the results of Figure 5, which show cell voltage losses (and thus impedance) dominated by the cathode, we can attribute the compressed semicircle feature to activation losses at the cathode. The span of the compressed semicircular feature is somewhat affected by the current density, with a span on the *x*-axis of ∼120 mΩ·cm^2^ for 0 mA/cm^2^, rising to a span of ∼132 mΩ·cm^2^ and 127 mΩ·cm^2^ for 100 and 250 mA/cm^2^ respectively. The span then decreases significantly for 500 mA/cm^2^, to about 106 mΩ·cm^2^.

**Figure 7:**
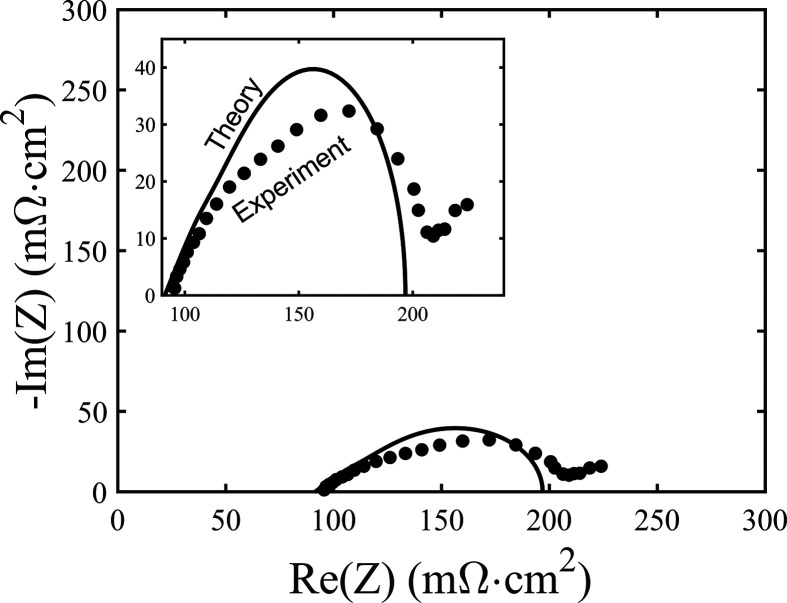
Comparison between the measured impedance of the cathode vs a Pd quasi-reference electrode at 0 mA/cm^2^ DC current (circular markers), and the developed linear impedance model (line). Inset shows a zoom-in view of the model and experimental results.

Generally, for systems with effectively planar electrodes such as PEM fuel cells, we would expect that electrode activation would be represented by a largely uncompressed semicircular feature, whose span would become markedly smaller with increasing current density. This is because the Faradaic resistance decreases with increasing current, according to the Butler–Volmer equation.^39^ However, for our membraneless H_2_–Br_2_ cell, we observe a strongly compressed semicircle and nonmonotonic span of the semicircle with increasing current density. The compression of the semicircle is expected behavior for a porous electrode, where the Faradaic resistance and double layer capacitance is distributed along the pore (Figure 1), which we will demonstrate below in Figure 7. A dependence of the semicircle span on the value of DC current is also expected and can be attributed to variations in local reactant concentration, reaction zone thickness, and current distribution in the porous electrode with DC current.^34^ For example, the effective Faradaic resistance and liquid phase resistance of the porous electrode are expected to be a function of DC current, as increasing current depletes the local reactant at the separator side of the porous electrode, likely elongating the reaction zone, varying the current distribution and therefore the effective liquid phase and Faradaic resistances.^33^ A detailed analysis of these latter effects, which would require relaxing the assumption of uniform electrolyte concentration in the porous electrode invoked in eq 4, is outside the scope of this study. Regarding features observed at the lowest frequencies and between around 220–275 mΩ·cm^2^ on the real axis in Figure 6, we hypothesize this is a mass transport impedance occurring in the porous cathode due to bromine concentration variations. Similar features observed at low frequencies were attributed to mass transport impedance by Huang et al., due to significant reactant concentration variations within the porous electrode occurring at low frequencies only.^34^

We now return to the model developed in the Theory section to confirm our interpretation of the main features in the Nyquist plot and to extract quantitative parameters governing the performance of the porous cathode. To our knowledge, our work contains the first comparison of a suitable impedance expression, which includes the porous electrode’s solid-phase resistance, to impedance data for high power density flow batteries. Figure 7 presents a comparison between the measured impedance of the cathode relative to the Pd quasi-reference electrode (circular markers), for the case of 0 mA/cm^2^ DC current density, and the best-fit model results, where the model impedance is given by eq 10. The independently measured or calculated parameters used in the theoretical prediction in Figure 7 include *l*_e_ = 0.93 mm, *A*_C_ = 0.75 cm^2^, *R*_L_^′^ = 2624 mΩ/cm, and *R*_S_^′^ = 856.8 mΩ/cm. *R*_L_^′^ was calculated using τ/*pA*σ, where σ is the electrolyte ionic conductivity of 710 mS/cm, *p* is the porosity of the uncompressed SGL 29AA carbon paper, *A*_C_ is the cell active area, and τ is the tortuosity calculated to be 1.12 via the Bruggeman relation. *R*_S_^′^ was calculated using ASR_S_/(*A*_C_*l*_e_) and the data from Figure 3 to obtain ASR_s_ = 59.8 mΩ·cm^2^ at *l*_e_ = 0.93 mm. The remaining parameters, *R*_f_^″^ and *C*_DL_^′^, and an external resistance, *R*_ext_, were obtained from a least mean squares fitting procedure of the model to experimental data. As described in the Theory section, our model assumes no reactant concentration variations, and thus during fitting we excluded the lowest frequencies which fell outside of the compressed semicircular feature and were attributed to mass transport impedance.

The results of the fitting are shown in Figure 7, and the best fit parameters were *R*_f_^″^ = 6.66 mΩ·cm^3^, *C*_DL_^′^ = 908 mF/cm^3^, and *R*_ext_ = 52.9 mΩ·cm^2^. *R*_ext_ represents the resistance external to the cathode, which for this measurement includes all resistances between the reference electrode and cathode, such as the resistance of the separator layer. As can be seen in Figure 7, the model results confirm that a compressed semicircle feature is expected for the cathode impedance. The shape of the compressed semicircle obtained experimentally is reasonably well-matched by that of the best-fit impedance using eq 10. We hypothesize that variations between the measured impedance and best-fit model results, where the measurement is slightly more compressed than the model result, in nonuniform cathode thickness in the battery, which is unavoidable in our membraneless design (see Methods section). To probe the extracted fitting parameters, we can compare the Faradaic resistance obtained here, *R*_f_^″^, to that of previous works with similar electrode materials. If we substitute *R*_f_^″^ into eq 11, we can obtain a volumetric exchange current density, *ai*_0_ = 1.93 A/cm^3^, which is near the value of 2.45 A/cm^3^ extracted by Chen et al., which was also for air-oxidized SGL carbon paper.^16^ Furthermore, the value of *C*_DL_^′^ is near the value of 698.8 mF/cm^3^ reported by Xie and Wang for oxidized carbon papers used for supercapacitors.^40^ Some deviation is seen between the theory and experiments, notably that the experimental semicircle is slightly more depressed than the theoretical one. Such features may be due to a not perfectly uniform current density in the porous cathode cross-section, as we expect the cathode to be slightly closer to the anode at the center of the active area than along the edges when the cell is under compression.

**Figure 8:**
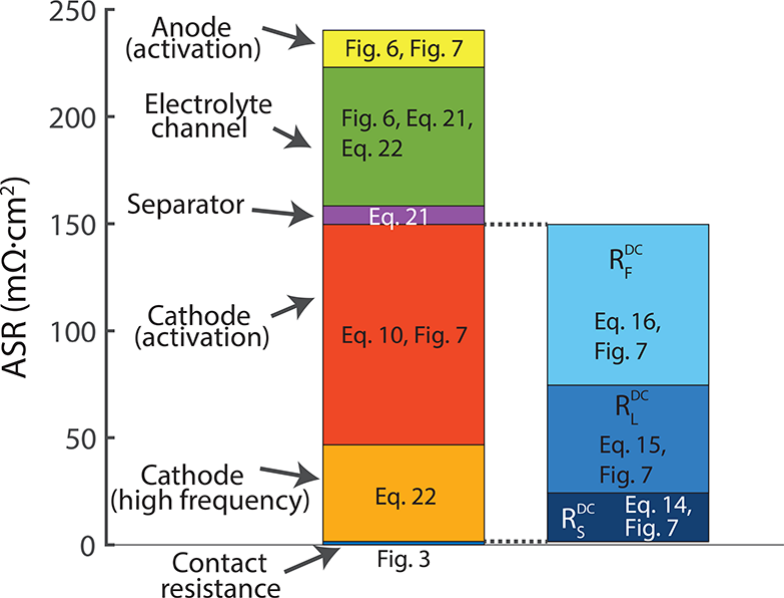
Breakdown of measured or calculated area specific resistance (ASR) from various sources within our custom-built membraneless hydrogen–bromine flow battery. The cathode ASR is broken down into activation and high frequency components (left bar graph) as well as DC Ohmic losses (right bar graph).

The data collected and analyzed between Figures 3–7, together with some simple calculations, can allow us to provide a detailed breakdown of resistive losses in the cell, which is summarized in Figure 8. First, we can decompose the resistances contributing to the high frequency resistance seen in Figure 6 of 120 mΩ·cm^2^: the Celgard separator, the electrolyte channel, and the high-frequency contribution of the cathode. We can calculate the ASR of the 3 M HBr-soaked Celgard using the expression
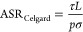
21where *L* is the thickness of the separator in the electric field direction of 25 μm, σ is the electrolyte ionic conductivity of 710 mS/cm, *p* is the separator porosity of 55%, and τ is the Celgard tortuosity of 1.35 using the Bruggeman relation, which yields 8.6 mΩ·cm^2^. Given the known solid and liquid-phase resistances of the porous cathode, ASR_s_ = 59.8 mΩ·cm^2^ (Figure 3) and ASR_L_ = 183.1 mΩ·cm^2^, we can calculate the high frequency resistance of the cathode as their parallel combination:^17^
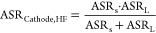
22yielding 45.1 mΩ·cm^2^. Further, as derived from the *y* intercept in Figure 3, there is a small contact resistance between the cathode carbon paper and the graphite current collector of 1.6 mΩ·cm^2^. Given the total high frequency resistance measured as 120 mΩ·cm^2^, this leaves 64.8 mΩ·cm^2^ for the electrolyte channel. The electrolyte channel’s nominal thickness is 650 μm, but its actual thickness in the battery is likely less due to the uncompressed cathode carbon papers intruding into this channel. We can calculate the effective thickness of the electrolyte channel using the expression for ASR of an open, electrolyte-filled channel of *L*/σ, which yields 460 μm. This implies that the cathode intruded ∼190 μm into the electrolyte channel, which is about the expected amount, as this represents approximately the difference between the uncompressed cathode thickness (930 μm) and the catholyte channel thickness (750 μm).

Together, the separator and electrolyte channel replace the membrane and are crucial in providing separation between the bromine stream and the anode. The ASR of a membrane in a high-power bromine-based battery with a 3 M HBr electrolyte was measured to be 62 mΩ·cm^2^,^16^ which is slightly lower than the combined ASR of our electrolyte channel and separator when filled with 3 M HBr of ∼73.4 mΩ·cm^2^. Thus, it will be important to reduce the ASR of the electrolyte channel substantially in future designs, for example by reducing its thickness from the nominal value of 650 μm used here to a thickness closer to the bromine boundary layer thickness in the electrolyte channel, which at the channel outlet of our prototype is expected to be approximately 200 μm.^26^ This could potentially reduce the ASR of the electrolyte to <30 mΩ·cm^2^.

The activation losses associated with the cathode are given by the real-axis span of the best-fit semicircle in Figure 7, which is 103 mΩ·cm^2^. Thus, the two largest sources of ASR for our membraneless cell are the cathode with 148.2 mΩ·cm^2^, including the high frequency and activation contributions, followed by the electrolyte channel with 64.8 mΩ·cm^2^. The cathode ASR can also be broken down into DC Ohmic and Faradaic losses, using eqs 14–20, which yields an *R*_S_^DC^ of 22.8 mΩ·cm^2^, *R*_L_^DC^ of 50.3 mΩ·cm^2^, and *R*_F_^DC^ of 75.1 mΩ·cm^2^ (Figure 8, right bar graph), which shows that improving the catalytic capability of the cathode (decreasing *R*_F_^DC^) is a potentially effective optimization path. We estimate that the anode activation loss is approximately 17 mΩ·cm^2^, which is the difference between the extracted cathode activation loss from Figure 7 and the span of the semicircle including both anode and cathode activation in Figure 6 at a 0 mA/cm^2^ current. Added all together, the total ASR of all components shown in Figure 8 is 240.1 mΩ·cm^2^, which is only slightly smaller than the slope of the polarization curve of Figure 4, representing the ASR of the entire cell, of 262 mΩ·cm^2^.

In conclusion, we here provided a detailed breakdown of resistances in a membraneless hydrogen–bromine redox flow battery, showing that the cathode dominated the overall cell resistance, and the resistance of the electrolyte channel was also significant. Future optimizations should thus focus on these two elements, which we believe can lead to significant improvement in achievable maximum power density. For example, the catalytic capability of the cathode can potentially be improved to reduce *R*_F_^DC^, and the electrolyte channel thickness can be minimized to the thickness of the bromine boundary layer in that channel.
